# Recent advances in two-dimensional ferromagnetism: strain-, doping-, structural- and electric field-engineering toward spintronic applications

**DOI:** 10.1080/14686996.2022.2030652

**Published:** 2022-02-17

**Authors:** Sheng Yu, Junyu Tang, Yu Wang, Feixiang Xu, Xiaoguang Li, Xinzhong Wang

**Affiliations:** a Institute of Information Technology, Shenzhen Institute of Information Technology, Shenzhen, China; b Institute for Advanced Study, Shenzhen University, Shenzhen, China; c Department of Physics and Astronomy, University of California, Riverside, CA, USA

**Keywords:** Two-dimensional ferromagnetism, magnetic tunnel junction, spin field-effect transistor, spin logic gate, strain engineering, doping engineering, 40 Optical, magnetic and electronic device materials, 105 Low-Dimension (1D/2D) materials < 100 Materials, 203 Magnetics/Spintronics/Superconductors < 200 Applications, 403 Electronic structure calculations < 400 Modeling/Simulations, 505 Optical/Molecular spectroscopy < 500 Characterization

## Abstract

Since the first report on truly two-dimensional (2D) magnetic materials in 2017, a wide variety of merging 2D magnetic materials with unusual physical characteristics have been discovered and thus provide an effective platform for exploring the associated novel 2D spintronic devices, which have been made significant progress in both theoretical and experimental studies. Herein, we make a comprehensive review on the recent scientific endeavors and advances on the various engineering strategies on 2D ferromagnets, such as strain-, doping-, structural- and electric field-engineering, toward practical spintronic applications, including spin tunneling junctions, spin field-effect transistors and spin logic gate, etc. In the last, we discuss on current challenges and future opportunities in this field, which may provide useful guidelines for scientists who are exploring the fundamental physical properties and practical spintronic devices of low-dimensional magnets.

## Graphical abstract

**Figure:**
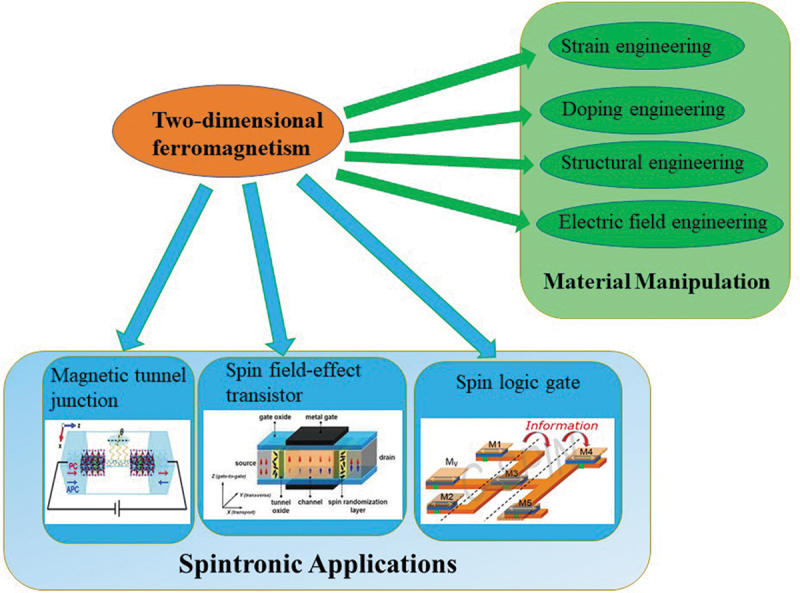

## 1. Introduction

Xu and Zhang et al. independently reported the ferromagnetic behavior in atomically thin layers of chromium germanium telluride (Cr_2_Ge_2_Te_6_) [1] and chromium triiodide (CrI_3_) [2] in 2017, using a polar magneto-optical Kerr effect microscopy technique. Subsequently, a wide variety of emerging two-dimensional (2D) materials with intrinsic magnetic ground states of ferromagnetism (FM) or anti-ferromagnetism (AFM) down to atomic-layer thicknesses have been discovered and predicted [3,4], as summarized in Table 1, for listing the values of their transition temperatures and coercive fields. Two-dimensional ferromagnetism was discovered in 2D materials with stable long-range magnetic ordering, exhibiting the ferromagnetic behavior at magnetic ground state. In comparison to the conventional bulk magnetic materials, the magnetic anisotropy plays a vital role for the stable magnetic order in 2D magnets. This can break the limitation of the Mermin–Wagner theorem that long-range magnetic order could not be induced in low dimensional isotropic systems with continuous symmetry at finite temperatures [5]. The magnetic exchange mechanisms in conventional 3D bulk magnets, including direct exchange interaction, superexchange interaction, Stoner-magnetism, and Ruderman–Kittel–Kasuya–Yosida (RKKY) mechanisms, can be found in various 2D magnets [6,7]. Also, some interesting exchange mechanisms, knows as super-spuerexchange, extended superexchange, and multi-intermediate double exchange, are newly discovered in 2D magnetic systems [7]. Meanwhile, distinct from their bulk materials, 2D magnets have an interesting thickness-dependent magnetism. For example, VSe_2_ and CrI_3_ monolayers demonstrated a ferromagnetic behavior, while their bilayer counterparts show interlayer-antiferromagnetism at low temperature [8,9]. The special attributes of 2D magnetism as compared to their bulk counterparts are briefly summarized below: (1) they show strong quantum confinement [10]; (2) they can be artificially integrated into heterostructure with arbitrary and flexible choices [11,12]; (3) their properties are thickness-dependent and highly anisotropic [13,14]; (4) they are the naturally perfect structure for magnetic and electronic tunneling effect [15,16], (5) they have large deformation and great endurance under external strain [17,18] and (6) they exhibit full tunability by external electric field, indicating a great potential for practical applications in voltage-controlled spintronic devices [19,20].

**Table 1.** The values of transition temperatures and coercive fields for emerging 2D magnetic materials

2D material	Ground state	Transition temperature (K)	Coercive field (T)
1L-FePS_3_	AFM	104 [173]; 118 [174]	N/A
1L-MnSe_2_	FM	300 [175]	0.15 [175]
1L-VSe_2_	FM	330 [30]	0.11 [30]
1L-Fe_3_GeTe_2_	FM	20 [66]	0.25 [66]
2L-Cr_2_Ge_2_Te_6_	FM	30 [1]	N/A
1L-CrBr_3_	FM	34 [176]; 27 [177]	0.005 [176]; 0.008 [177]
2L-CrBr_3_	FM	36 [177]	0.01 [177]
2L-CrCl_3_	AFM	16 [177]	0.98 [177]
2L-CrI_3_	AFM	45 [177]	0.8 [177]; 0.7 [127]
1L-CrI_3_	FM	45 [2]	0.05 [2]; 0.13 [127]

Spintronics, exploiting the spin-polarized electrons/holes as the information carrier, has quickly attracted a great deal of attention in the field of next-generation nanoelectronics. Compared to conventional nanoelectronics, a low energy consumption could enable the switching of the spin state with a much faster operation in spintronic devices. Therefore, spintronics has been developed into the most promising technology aiming to achieve information storage, transmission, and processing. 2D materials have drawn tremendous attention in spintronics due to their distinctive spin-dependent properties, such as the long spin relaxation lengths and times in graphene [21,22] and the stable spin-valley locking in transition metal dichalcogenides [23,24]. Moreover, the heterostructures by van der Waals engineering offer an unprecedented platform for effectively regulating interfacial magnetic order by magnetic proximity effect, which can break through the limitation of individual 2D magnet [25]. However, several challenges remained to be solved in 2D spintronics. Firstly, most 2D magnetic materials used in spintronics have low Curie temperature (*T*_c_) far below room temperature, as shown in Table 1. Secondly, precise spin-state detection and manipulation have not been achieved yet, which means the inability of spin-information transmission and impossibility for its spintronic applications. Moreover, the polarization efficiency of the spin injection and gating are urgently in need of improvement. Consequently, the currently reported magnetoresistance in 2D spin-transport devices are still very low, which cannot be used for the basic logic unit in very-large-scale integration (VLSI).

With the rapid development of 2D spintronics, it is urgent to review on the latest studies on current 2D magnetic materials and their applications. This review will focus on the recent scientific endeavors on the various engineering strategies on 2D ferromagnets, such as strain-, doping-, structural- and electric field-engineering, toward practical spintronic applications, including spin tunneling junctions, spin field-effect transistors, and spin logic gate, etc. We have chosen strain-, doping-, structural- and electric field-engineering as the highlighted subtopics because all of strategies are proven to be very effective approaches to tune the individual properties of atomic layers. Also, these engineering methods have been proficiently achieved in the experiments, such as substrate bending for strain-engineering [26], ion liquid gating for doping-engineering [27], pick-up transfer technique for integrating atomic hetero-structures [28] and dual gate technique for electric-field engineering [29] and so on. The various engineering approaches discussed in this review have induced multiple spintronic device-favored mechanisms, for example, the enhanced magnetic exchange interactions and elevated Curie temperature by strain-engineering, and the Stoner-ferromagnetism by electrostatic-doping, both of which are of great importance to the operational reliability of spintronic device at room temperature. Also, both the reduced spin relaxation and large carrier mobility induced by electrostatic-doing, and efficient spin-charge conversion and charge transport via proximity effect by structural engineering play a vital role in the performance of spin field-effect transistor. Meanwhile, the stronger spin filtering effect and enhanced magnetoresistance by using van der Waals heterostructure are also very favourable for the performance of magnetic tunnel junction and spin valve device. In the final section, we pointed out some current challenges and future opportunities in this field aiming to achieve practical applications. We attempt to summarize the underlying physical mechanism and issues for past various independently experimental and theoretical studies on the associated 2D magnetic nanostructures. We strongly believe that our comprehensive overview and general insight into 2D ferromagnetism can provide useful guidelines for exploring both novel engineering approaches and future candidates for spintronic devices of 2D magnets.

## 2. Manipulation of ferromagnetism in 2D materials

### 2.1. Strain engineering

The strain effect is inevitable in the experimental synthesis process for integrating 2D magnets on the substrate, for example, transferring CrI_3_ or Cr_2_Ge_2_Te_6_ atomic layers on SiO_2_/Si substrate by exfoliation [1,2], or fabrication of NbTe_2_ and VSe_2_ on SiO_2_/Si substrate by CVD/MBE [30,31]. The lattice mismatch between 2D magnets and the substrate can induce the interfacial strain, which can moderately modulate the performance of 2D magnets. Strain engineering has been demonstrated as a powerful technique for tuning the electronic properties of 2D materials, such as band gap, effective mass and carrier mobility (e.g. using substrate lattice mismatch [32,33] or substrate bending [18]). Typically, in contrast to the bulk crystals, 2D materials demonstrate remarkable endurance under much larger strains [17,18]. For instance, single atomic layer FeSe and MoS_2_ can undergo external strains up to 6% and 11%, respectively [34–36]. Also, the reduced dimension in 2D layered structure endows a smaller young modulus and softness. For example, it was reported that chromium trihalides are extremely soft with the small 2D Young’s modulus of 24, 29 and 34 N*/*m for monolayer CrI_3_, CrBr_3_ and CrCl_3_ by DFT calculations, respectively [37]. The large strain endurance and soft nature of 2D layered structure imply that their magnetic properties can be effectively manipulated by strain engineering.

**Figure 1.**
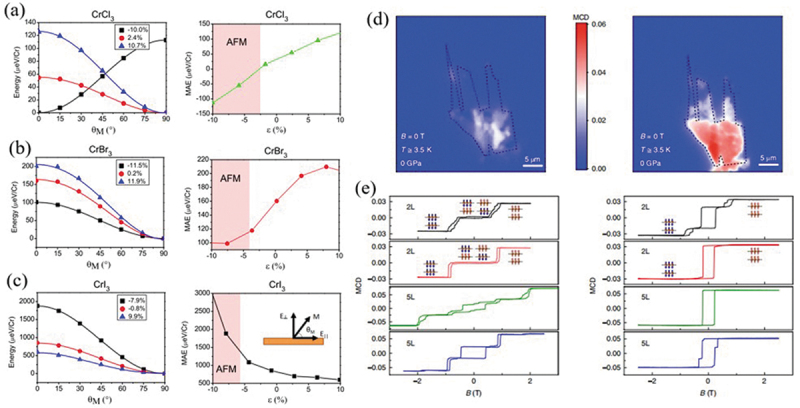
Change in energy with respect to the magnetization angle *θ*_*M*_ and MAE with respect to strain for (a) CrCl_3_, (b) CrBr_3_ and (c) CrI_3_. Reproduced with permission from [37], Copyright 2018, American Physical Society. (d), MCD image of the CrI_3_ flake before (left) and after (right) applying a pressure of 1.8 GPa. (e) Magnetic field dependence of MCD at 3.5 K for two 2-layer (2 L) and two 5-layer (5 L) regions before (left) and after (right) applying pressure. Reproduced with permission from [42], Copyright 2018, Nature.

Strain can modulate the structural anisotropy, and consequently altering the magnetic anisotropy property by effectively tuning the magnetic exchange interactions. Webster et al. [37] reported a theoretical study on the strain dependence of magnetic anisotropy energy in 2D monolayer chromium trihalides of CrCl_3_, CrBr_3_ and CrI_3_, respectively (Figure 1(a–c)). They demonstrated that the magnetic anisotropic energy increases when a compressive strain is applied on CrI_3_ monolayer, while an opposite trend is observed in the other two compounds. In particular, the magnetic anisotropic energy of single layer CrI_3_ can be enhanced by 47% under a compressive strain of *ε *= 5%. Xu et al. demonstrated that, based on their first-principles calculations, an in-plane 90° rotation of the magnetic easy axis can be reversibly induced by applying the ferroelastic strain on chromium sulfifide halides CrSX (X = Cl, Br, I) monolayers [38].

The lattice deformation induced by strain can alter the exchange coupling strength between magnetic ions, correspondingly change the magnetic thermal stability and the phase transition temperature. Zhou et al. [39] showed NbS_2_ and NbSe_2_ monolayers can be magnetized with high Curie temperatures of 387 and 542 K under 10% biaxial tensile strain. This ferromagnetic character can be attributed to the competitive effects between through-space interaction and through-bond interaction. Miao et al. [40] suggested that CrOCl monolayer could be feasibly prepared from their bulk counterparts because its exfoliation energy is only around 0.208 J/m^2^, which is only two-third of graphite. Moreover, the *T_c_* of CrOCl monolayers can be significantly enhanced from 160 to 204 K under 5% biaxial tensile strain. Huang et al. [41] predicted that room-temperature ferromagnetism could be achieved under a tensile in-plane strain of 4% in monolayer CrWI_6_ and CrWGe_2_Te_6_. The magnetic phase transition can also be induced by external strain, which can induce the multiple crystal-lattice effects, such as structural phase transition, layer-displacement, structural deformation and so on. Li et al. [42] modulated the stacking order by a monoclinic-to-rhombohedral change via applying strong hydrostatic pressure on atomically thin CrI_3_ with 2D van der Waals structures. They observed an irreversible phase transition from interlayer AFM to FM in atomically thin CrI_3_ by magnetic circular dichroism microscopy (Figure 1(d,e)). Leon et al. [43] demonstrated that a phase transition from AFM to FM in bilayer CrI_3_ can be induced by the in-plane compressive strain. They associated that behavior with the relative inter-layer displacement induced by external strain. Li et al. [44] reported that the biaxial strain can induce a transition from FM to the AFM state in Mn-doped Silicene with the strain-tunable magnetic exchange couplings.

### 2.2. Doping

#### 2.2.1. Magnetic doping

As an effective tool in functionalizing 2D systems, defect doping can induce magnetism in an atomic layered structure, where the long range magnetic order arises from the presence of single-atom defects in combination with an intrinsic discriminating mechanism in sublattices, in confirmation with Lieb’s theorem [45,46]. 2D transition-metal dichalcogenides (TMDs) provide the perfect platform with appropriate defective localization for using various doping approaches, including introducing vacancy, atomic substitution and displacement. The TMDs introduced by transition metal atoms (e.g. Fe, Co, Ni, Mn, etc.) with large magnetic moment have been proposed as diluted magnetic semiconductors, which may have promising applications in 2D spintronics [47–55]. Mishra et al. [50] reported a theoretical investigation on long-range FM ordering in Mn-doped MoS_2_, MoSe_2_, MoTe_2_ and WS_2_ by substituting Mo or W sites by Mn. Their first-principles calculations demonstrated an AFM exchange between the Mn *d* states and the *p* states of the chalcogen atoms, which can effectively regulate the long-range FM exchange of Mn atoms. Cheng et al. [51] presented a comprehensive first-principles study of MoS_2_ monolayer doped by various transition metals. They observed ferromagnetism in MoS_2_ monolayer doped by Mn, Zn, Cd and Hg for concentration of 6.25%, while for Co and Fe dopants, AFM is observed due to Jahn-Teller distortions. Kanoun et al. [52] investigated the electronic and magnetic properties of monolayer MoTe_2_ by introducing Mo vacancy or substituting Mo by various 3d transition metals by using first-principles calculations. They observed stable magnetic moment for all these cases, except for Ni defect. Zhao et al. [53] observed ferromagnetism in 1 T-ZrSe_2_ monolayer by V, Cr, Mn, and Fe doping. They found that the magnetic moments of dopants have been significantly increased after including Hubbard potential *U_eff_* during using density functional theory, indicating a transition from the low to high spin state. In addition to TMDs, diluted magnetic semiconductors have also been predicted by other 2D materials with magnetic dopants. Wu et al. [54] theoretically demonstrated the room-temperature ferromagnetism with Curie temperature of 629 K in Fe-doped SnS_2_ with magnetic momentum of 2.0 *μ*_*B*_ per Fe atom. They observed the long-range ferromagnetism with intralayer sites occupied by the dopants, while it is paramagnetic with Fe fixed at interlayer sites. Zberecki et al. [55] predicted the magnetism in TMs-doped 2D honeycomb structures of III–V binary compounds (i.e. GaN, AlN and InN). Another useful strategy for magnetic doping is through introducing atomic vacancies. Yazyev et al. [56] demonstrated that, based on first-principles calculations, the itinerant magnetism can be induced in graphene with magnetic moments of 1 *μ*_*B*_ per hydrogen chemisorption defect and 1.12–1.53 *μ*_*B*_ per vacancy defect for various defect concentrations. The type of magnetism (i.e. FM or AFM) is determined by whether introducing truly disordered system during defect engineering. Jang et al. [57] showed that Fe_3_GeTe_2_ is not FM but intrinsically has AFM ground state by using first-principles calculations. They also proposed that the hole doping induced by Fe deficiency can induce AFM to FM transition.

**Figure 2.**
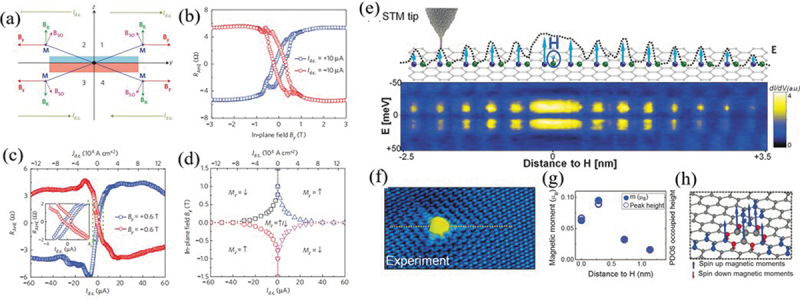
(a) Schematic of the four stable magnetization states when passing a large d.c. current and applying an in-plane external magnetic field. The effective spin–orbit field induced by the d.c. current and the anisotropy field are both considered. (b) The AHE resistance as a function of the in-plane external magnetic field when passing a constant d.c. current at 1.9 K. (c) Current-induced magnetization switching in the Hall bar device at 1.9 K in the presence of a constant in-plane external magnetic field. (d) Phase diagram of the magnetization state in the presence of an in-plane external magnetic field *B*_*y*_ and a d.c. current. Reproduced with permission from [59], Copyright 2014, Nature. (e) Conductance map [dI/dV(x,E)] along the dashed line in (f). (f) STM topography of a single H atom on graphene. (g) Comparison between DFT calculations for the local magnetic moment and the height of the occupied projected DOS (PDOS) peak. (h) Calculated magnetic moments induced by H chemisorption. Reproduced with permission from [64], Copyright 2016, Science.

It is noteworthy that the theoretical studies mostly focused on constructing 2D magnetic lattice at the atomic-scale with enhanced magnetic moments, stronger exchange interactions and elevated Curie temperatures, while the experimental studies turned their attentions to spin transport properties, including confined spin relaxation, effective spin-charge conversion, large magnetoresistance, high carrier mobility and so on. Apparently, experimental concentrations on the spin transport properties in 2D systems are aiming to develop practical spintronic devices, such as spin field transistors and magnetic tunnel junction. Experimental studies on magnetic doping in 2D layered structures and van der Waals (vdW) heterostructures have been made great progress. Pi et al. [58] reported the effects of surface gold deposition on spin transport in graphene by fabricating a spin valve device at low temperature. They showed that the charge impurity scattering plays a minor role in spin relaxation while a significant enhancement of spin lifetime from 50 to 150 ps was observed with increased gold coverage. Wang’s group [59] experimentally demonstrated an effective regulation on magnetization via giant spin-orbit torque induced by an in-plane current in an epitaxial Cr-doped topological insulator film on GaAs substrates. The effective field to the in-plane current amplitude ratio and the spin Hall angle have been measured as several orders of magnitude larger than that in undoped heavy metal/ferromagnetic heterostructures (Figure 2(a–d)). Furthermore, this team [60] reported an effective electric field control of spin-orbit torque in a Cr-doped (Bi_0.5_Sb_0.42_)_2_Te_3_ thin film by using the spin field-effect transistor. The SOT intensity can be regulated by several orders of magnitude by varying the gate voltage with an effective magnetization switching. Nie et al. [61] fabricated the unique Mn_*x*_Ge1-_*x*_ nanomeshes with controllable Mn-doping density by nanosphere lithography. The superconducting quantum interference device demonstrated a high *T_c_* above 400 K, which indicated a significant enhancement of magnetic exchange couplings as a result of quantum confinement effect in unique nanomeshed structure. Furthermore, a giant magnetoresistance of ~8,000% is realized at low temperature of 30 K in Mn_0.03_Ge_0.97_ nanomesh. Li et al. [62] synthesized Fe-doped SnS_2_ bulk crystals with different Fe contents via a direct vapor-phase method, and then obtained their monolayer flakes via mechanical exfoliation. The electric transport measurement showed that the field-effect transistor based on exfoliated Fe_0.021_Sn_0.979_S_2_ demonstrate a high on-off ratio of ~1.2 × 10^6^ and carrier mobility of ~6.1 cm^2^ V^−1^ s^−1^. The magnetic measurements exhibited FM ground state with a remarkable perpendicular anisotropy at 2 K and a Curie temperature of ~31 K in Fe_0.021_Sn_0.979_S_2_ nanosheet. Tokmachev et al. [63] fabricated layered silicene doped with rare-earth ions (Gd^3+^ and Eu^2+^) ranging from the bulk down to one monolayer. They demonstrated a thickness-dependent magnetism that the bulk is antiferromagnetic while its ultrathin layers have intrinsic 2D in-plane ferromagnetism.

The stable long-range magnetic ordering and local magnetic moments can be also induced in non-magnetic 2D materials with non-magnetic dopants. Only one state of π-state couple near the Fermi level can be occupied by single electron due to the electrostatic Coulomb repulsion, which can extend the spatial range of the direct magnetic coupling by several nanometers. This can break the limitation of the localized magnetic coupling effect with relatively short spin exchange distance, which is the prerequisite of the conventional magnetic doping with metallic ions. González-Herrero et al. [64] demonstrated that a spin polarization and stable magnetic ordering can be induced in hydrogen-adsorbed graphene by both scanning tunneling microscopy experiments and first-principles calculations. They observed that the magnetic moments are essentially localized between the carbon atoms in graphene-sublattice in an opposite direction with the chemisorbed-hydrogen locations (Figure 2(e–h)). They also demonstrated a remarkably precise control of magnetic momentum by selectively removing chemisorbed-hydrogen atoms in the arbitrary sublattice.

#### 2.2.2. Electrostatic doping

In contrast to magnetic doping, electrostatic doping can also be used to control the carrier concentrations of the ferromagnets by virtue of without introducing defects. In comparison to 3D bulk materials, atomic thin 2D materials take great advantage of naturally large surface-area-to-volume ratio for charge doping engineering. By virtue of their atomic thickness, the gating technology can tune the carrier doping concentrations up to 10^13^ cm^−2^ in MoS_2_ and WSe_2_ monolayer [29,65] and 10^14^ cm^−2^ in 2D Fe_3_GeTe_2_ thin film [66], and up to the order of 10^14^ cm^−2^ in graphene [26,27]. Hence, gate-tunable doping has become an effective way to modulate the electronic state around the Fermi energy.

**Figure 3.**
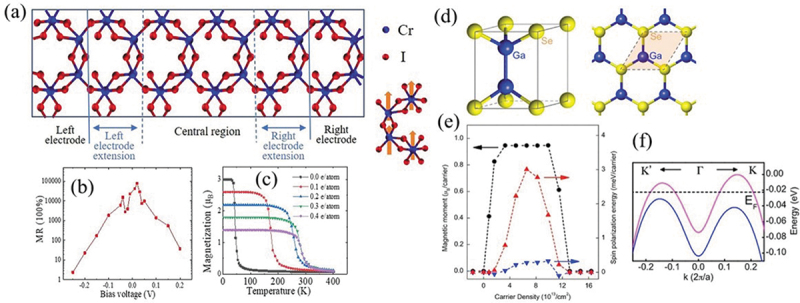
(a) The device model based on the hole-doped 1 L-CrI_3_. (b) The magnetoresistance vs. the bias voltage. The hole doping density is 0.05 e/atom (9.43 × 10^13^ cm^−2^). (c) The magnetic moment as a function of temperature under various hole doping density. Reproduced with permission from [69], Copyright 2021, Elsevier. (d) The crystal structure of monolayer GaSe. (e) Carrier density dependence of spin magnetic moment per carrier and spin-polarization energy per carrier in the out-of-plane spin-polarized ferromagnetic state. (f) Band structures along high symmetry directions at carrier density of 7 × 10^13^/cm^2^. Reproduced with permission from [45], Copyright 2015, American Physical Society.

Błonski et al. [67] reported the stable ferromagnetism can be induced in graphene by doping with graphitic, pyridinic, and chemisorbed nitrogen. Graphene doped below 5 at.% of nitrogen shows dominant diamagnetic; while if the doping concentration is above 5 at.%, it demonstrated a transition to ferromagnetism with *T_c_* of 69 K. Their first-principles calculations showed that graphitic nitrogen atoms play the predominant role in inducing Stoner-magnetism, indicating a spin-polarized electrons near Fermi levels. Zhang et al. [68] showed that electrostatic doping can endow single layer PdSe_2_ with half metal Stoner-ferromagnetism and high *T_c_* above room temperature. Yu et al. [69] and Wang et al. [70] independently predicted that the hole doping can cause half-metallicity and nearly 100% spin-polarization in monolayer CrI_3_ by using first-principles calculations. Meanwhile, the magnetoresistance can reach over 10^6^% via hole doping (Figure 3(b)). Moreover, the stability of itinerant ferromagnetism can be significantly strengthened by hole doping so that a room temperature of *T_c_* can be achieved with the doping concentration of 8.49 × 10^14^ cm^−2^ (Figure 3(c)).

The electrostatic gating on non-magnetic 2D materials could also induce the large electronic density of states near the Fermi level with a strong spin-exchange, resulting in spontaneous spin polarization and the stable itinerant ferromagnetism. Cao et al. [71] and Wu et al. [72] separately predicted that hole doping can induce itinerant ferromagnetism and half-metallicity in single layer GaSe. The doping system exhibits nearly constant magnetic moment per carrier around 1.0 µB with the doping concentration between 3 × 10^13^/cm^2^ and 1 × 10^14^/cm^2^. The itinerant ferromagnetism is attributable to the large exchange splitting between spin-up and spin-down electrons at top of valence band and the high electronic density of states with a remarkable van Hove singularity near Fermi level [45] (Figure 3(d–f)). The family of group IIIA metal-monochalcogenide MX (M = Ga, In; X = S, Se, Te) single layers [73] and 2D holey C_2_N crystals [74] have also been demonstrated to possess high density of states at band edges, leading to Stoner-ferromagnetism via carrier doping engineering. Fu et al. [75] predicted that in buckled phosphorene and arsenene, hole doping can induce Stoner-ferromagnetim with a plateau magnetic moment per carrier around 1.0 µB in a broad range of doping concentrations between 3 × 10^13^/cm^2^ and 5.6 × 10^14^/cm^2^. They also demonstrated the strain-tunable Stoner-ferromagnetism by altering the band orders at top of valence band.

### 2.3. Structural engineering

Most of spin logic devices, including spin field-effect transistor, spin valve and magnetic memory, require a high on-off ratio, which means a great distinction in spin transport properties between spin-up and spin-down electrons. However, this is a challenge for graphene and even for most 2D insulators [76,77]. One approach to overcome this obstacle is to interface 2D materials with 3D magnets (i.e. exfoliation). In comparison to this approach by using 3D magnets, a 2D van der Waals heterostructure has the following advantages: (i) The interlayer twisting angle and the various stacking orders endow the heterostructure with richer properties by the arbitrary choice of direct- or indirect-bandgap, which can broaden the applicability for high-performing magnetic devices [78]. (ii) There is no request for the lattice matching, accordingly keeping their pristine atomic layered structure without chemical bonding and interfacial damage [12]. (iii) The diverse and flexible choices of 2D magnetic materials can enable the precise control of the fabrication process and comprehensive characterization of the heterostructure [11]. (iv) The proximity coupling effect at atomically sharp interfaces between vdW materials and magnetic substrates could effectively tune the spin-related properties in pristine layer, including spin-orbit coupling, spin polarization and spin transport [79]. (v) The heterostructure can modulate the magnetic ions in 2D diluted magnetic semiconductors, and consequently raising the temperature at which the quantum anomalous hall effect (QAHE) can be observed [80]. (vi) Multiple interfacial mechanisms, including charge transfer, band alignment, symmetry breaking, orbital hybridization and layer polarizability, have been proposed as very effective tools to modulate the magnetic properties [81].

The 2D heterostucture based on 2D non-magnetic material/magnets can have promising valleytronic applications. Valleytronics is a novel practical approach for information processing and storage by utilizing the spin and valley degrees of electron freedom. The 2D heterostructure can lift the valley degeneracy and produce the valley splitting by interfacial effect and ultimately induce a robust and nonvolatile valley polarization, which is of great importance to the creation, conduction and storage of magnetic informations. Sabirianov et al. [82] fabricated the heterostructure of WS_2_ monolayer and ferromagnet EuS. They found that the giant valley splitting of 16 meV can be induced by magnetic proximity effect, which is two orders of magnitude larger than that obtained by applying external magnetic field. Their first-principles calculations revealed that the sign reversal between WSe_2_/EuS and WS_2_/EuS is attributed to the different atomic termination at EuS surface sites. Kang et al. [83] performed first-principles calculations and observed a considerable valley splitting energy of 376 meV at the valance band of 2D WS_2_/h-VN heterostructure, which corresponds to an effective Zeeman magnetic field of 2703 T. Feng et al. [84] also used first-principles calculations to demonstrate a large valley spitting of 214 meV at the valence band of monolayer WS_2_ on the MnO(111) surface, which is equivalent to a Zeeman magnetic field of 1516 T. Gong et al. [85] observed a large valley splitting over 30 meV at the conduction band of WSe_2_ monolayer in 2D MnPSe_3_/WSe_2_ heterostructure.

Additionally, structural engineering can also endow the significant enhancement of magnetic anisotropy, exchange interactions and Curie temperature as a result of interfacial effect. Yamasaki et al. [86] fabricated the vdW heterostructure composed of Cr_1/3_TaS_2_ and ferromagnet Fe_1/4_TaS_2_ with a native T_2_O_5_ tunnel barrier in between by employing a dry-transfer method. Cr_1/3_TaS_2_ exhibited a great in-plane magnetic anisotropy and a maximum tunnel magnetoresistance (TMR) of 13% versus bias voltage. Hou et al. [87] showed that a thickness-dependent spin splitting of the Dirac cone at Bi_2_Se_3_ films surface can be realized *via* forming the topologically nontrivial CrI_3_/Bi_2_Se_3_/CrI_3_ heterostructure by their first-principles calculations. Dong et al. [88] showed that the magnetic anisotropic energy of Cr_2_Ge_2_Te_6_*/*PtSe_2_ bilayer heterostructure can be enhanced by 70% compared with single layer Cr_2_Ge_2_Te_6_ by using first-principles calculations. They attributed this great enhancement to both the presence of Dzyaloshinskii–Moriya interaction and single ion anisotropy. Additionally, the Curie temperature is also increased significantly above 600 K by DFT calculations.

**Figure 4.**
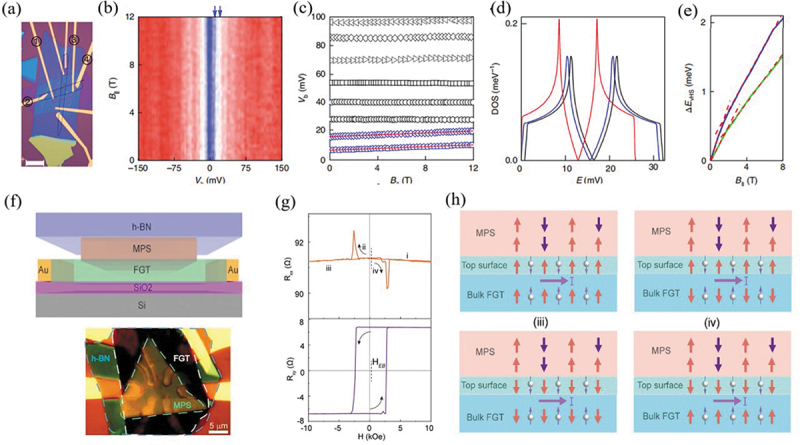
(a) An optical micrograph of the investigated device. (b) Differential tunneling conductance *G* as a function of *B_‖_* and *V_b_* (*V*_g_ = 0 V). The color scale is blue to white to red, 6 nS to 12 nS to 19 nS. (c) Bias position of the step-like features in *G* as a function of *B_‖_*. (d) Calculated magnon density of states for *T* = 10 K, *B* = 0 T (blue line), *T* = 10 K, *B* = 6.25 T (black line), and *T* =* T_c_, B* = 0 T (red line). The same calculations provide *T_c_ *= 88 K. (e) Calculated changes of the position of the van Hove singularities in magnon density of states. Reproduced with permission from [90], Copyright 2018, Nature. (d) as a function of magnetic field for temperatures close to *T_c_*. (f) Schematic illustration and optical image of the vdW heterostructure of MPS/FGT. (g) Magnetic field dependence of *R*_xx_ and *R*_xy_ in the MPS/FGT heterostructure at 10 K with a positive shift of HEB = 160 Oe at a cooling field of HFC = −10 kOe. (h) Schematic diagram of the spin polarization and magnetization at interface and bulk FGT. Reproduced with permission from [92], Copyright 2020, American Chemical Society.

Structural engineering can also effectively improve the spin transport of 2D magnets, via enhancing spin injection, polarization and magnetoresistance as well as introducing multiple magnetoresistance states, all of which are aiming towards the practical goals of spin valve, spin field-effect transistor, magnetic tunnel junction and so on. Gurram et al. [89] experimentally reported that graphene can have a giant spin-injection efficiency and large spin polarization under bias voltage in bilayer h-BN/graphene/h-BN heterostructure by using four-terminal structure with ferromagnetic electrodes. Ghazaryan et al. [90] studied tunneling effect in graphene/CrBr_3_/graphene, where thin ferromagnetic CrBr_3_ are tunneling barriers and graphene are used as electrodes. They demonstrated that the spin injection and spin polarization through thin ferromagnet CrBr_3_ barriers are mainly associated with the momentum conservation condition, which is manipulated by magnon emission at low temperature (Figure 4(a–e)). Albarakati et al. [91] demonstrated unusual antisymmetric magnetoresistance effect in vdW Fe_3_GeTe_2_/graphite/Fe_3_GeTe_2_ heterostructure devices. When the magnetic momentum in two Fe_3_GeTe_2_ flakes are antiparallel, the magnetoresistance of these devices possesses distinct high- or low-resistance states, while it adopts an intermediate state in case of parallel magnetization in these two ferromagnets. Based on their first-principles calculations, the three-resistance characteristic was attributable to spin momentum locking at the graphite/FGT interface as a result of Rashba-split in 2D electron gas. Hu et al. [92] theoretically studied the spin transport property in a vdW heterostructure of MnPS_3_/Fe_3_GeTe_2_, where MnPS_3_ is an intrinsic antiferromagnet while Fe_3_GeTe_2_ is a ferromagnet. The magnetoresistance exhibits three spin-transport states of high-, intermediate- and lowlevels. This phenomenon is attributed to a desynchronized phase during magnetic switching at MnPS_3_/Fe_3_GeTe_2_ interface under external magnetic field (Figure 4(f–h)). Lin et al. [93] investigated the spin transport property of the spin-valve device based on Fe_3_GeTe_2_/MoS_2_/Fe_3_GeTe_2_ vdW heterostructures. The current–voltage curve illustrates a perfect linear characteristic, indicating a good Ohmic contact at Fe_3_GeTe_2_ /MoS_2_ interfaces. The magnitude of the magnetoresistance is measured as 3.1% at 10 K and it decreases monotonically as the increasing temperature up to 200 K. Khan et al. [94] reported on the spin transport properties of the spin valve heterostructure interfacing by bilayer graphene and single-layer MoSe_2_ with metallic cobalt and nickel–iron alloy as the ferromagnetic electrodes. The device exhibited a positive MR, i.e. ~ 1.71% and ~ 1.86% at low temperature before and after annealing, respectively.

With the fast development of synthesis technology for 2D heterostructures, proximity effect could be employed to effectively modulate the multiple characteristics of pristine layer. A given 2D material can be endowed with superconducting, magnetic, or topologically states by proximity effect. For instance, although pristine graphene has negligible SOC strength, it can realize efficient spin-charge conversion and charge transport via proximity effect with the adjacent ferromagnets [95–97]. Mendes et al. [95] fabricated graphene on top of thin ferromagnetic insulator yttrium iron garnet (YIG) film by large-area CVD method. They observed that the spin current can be converted into charge current by the inverse Edelstein effect.

**Figure 5.**
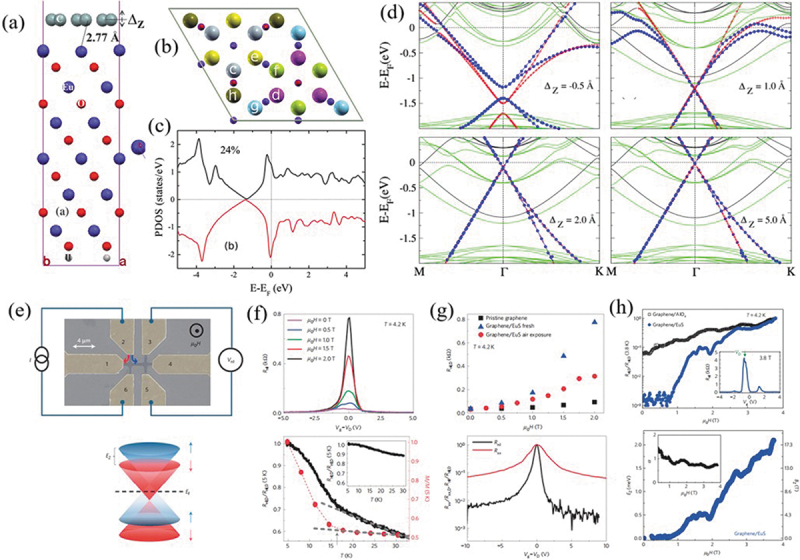
(a) Side view and (b) six sublattices of the calculated crystalline structures for graphene on top of a six bilayer EuO film. (c) Total density of states of p_z_ orbitals of graphene. (d) Band structures for graphene on EuO with graphene shifted by different distances. Reproduced with permission from [100], Copyright 2013, American Physical Society. (e) Top panel: a false-colored device image taken by a scanning electron microscope. Bottom panel: a schematic drawing of the Zeeman splitting of the Dirac cone in graphene and the spin-up hole-like and spin-down electron-like carriers at the charge neutrality point. (f) Top panel: Non-local resistance *R*_nl_ as a function of gate voltage *V_g_* under dierent *µ_0H_* for a CVD-graphene/EuS device at temperature *T*. Bottom panel: Comparison of *R*_nl,D_ versus *µ_0H_* curves for the graphene device before (pristine) and after EuS deposition. (g) Top panel: Comparison of the temperature dependence of *R*_nl,D_ and of *M* of the graphene/EuS heterostructure. Bottom panel: Comparison of the normalized non-local resistance and longitudinal resistance in graphene/EuS. (h) Top panel: Field dependence of *R*_nl,D_ in graphene/EuS versus that in graphene/AlO_x_. Bottom panel: Quantitative estimation of the Zeeman splitting energy *E_z_* in the presence of EuS. Reproduced with permission from [101], Copyright 2016, Nature.

Graphene can also gain magnetism and spin transport by magnetic proximity effect [98,99]. Wang et al. [99] demonstrated the anomalous Hall effect with Hall conductance of ~2e^2^/h at low temperature of 2 K in graphene when adhering to atomically thin YIG ferromagnet. Their observed the long-range ferromagnetic order induced by proximity effect in graphene/YIG heterostructure. The significant enhancement of spin-orbit coupling strength in comparison to the pristine graphene has been also demonstrated. Yang et al. [100] found that, based on their first-principles calculations, the spin-polarization ratio of *p_z_* orbitals in graphene is about 24%, together with a large spin exchange-splitting energy of about 36 meV due to C-*p_z_* and Eu-*4 f* interaction in graphene/EuO heterostructure. Meanwhile, they demonstrated that the stacking order and interlayer distance of the hetero-atomic layers mainly determined the position of the Dirac cone (Figure 5(a–d)). Wei et al. [101] reported a substantial enhancement of the spin signal by several orders of magnitude stemming from the Zeeman spin Hall effect in graphene induced by adjacent ferromagnetic insulator (EuS). They also demonstrated that the strong exchange magnetic field can lift the ferromagnetic ground-states degeneracy of Dirac electrons and create the spin-polarized valley-singlet state (Figure 5(e–h)). Lazić et al. [102] showed that the magnetic proximity tuned by gating effect can occur in 2D heterostructures, i.e. Co/bilayer graphene, Co/BN/graphene, and Co/BN/benzene, due to the vdW bonding interaction. They pointed out that the van der Waals bonding is required for both modulating the spin polarization and enabling its sign reversal. Zhong et al. [103] demonstrated an effective regulation of the valley dynamics in WSe_2_ via switching the CrI_3_ magnetization in CrI_3_/WSe_2_ heterostructure. Their photoluminescence measurement at low temperature reveals zero-field valley splitting of ~3.5 meV, which is equivalent to an effective 13 T magnetic field due to the presence of ferromagnetic substrate CrI_3_. Wu et al. [104] found that the magnetic proximity effect can result in a remarkable Zeeman splitting equivalent to the presence of magnetic field with magnitudes of several hundreds of tesla in graphene adhering to ferromagnetic EuO substrate. They also demonstrated a magnetic state transition of the first Landau level from ferromagnetism to a canted antiferromagnetism via applying the perpendicular magnetic field. Tong et al. [105] theoretically studied the magnetic proximity effect in vdW moire´ superlattice formed by semiconductor monolayer BAs and ferromagnetic monolayer CrI_3_ by first-principles calculations. Their study showed that the interlayer atomic configuration plays a very important role in the magnetic proximity effect due to the spin-dependent interlayer hopping. Karpiak et al. [106] observed that the spin transport and precession in graphene can be effectively modulated by adjacent ferromagnetic Cr_2_Ge_2_Te_6_ with strong anisotropy via magnetic proximity effect. The lifetime for perpendicular spins is measured as 3.9 times larger than that of the in-plane counterpart, indicating the strong anisotropy of spin texture spectroscopy in graphene. Recently, Zhang et al. [107] showed that the ferromagnetism of Fe_3_GeTe_2_ thin films can be modulated by interfacing with atomically thin FePS_3_. The Curie temperature of Fe_3_GeTe_2_ is improved by 30 K in FePS_3_/Fe_3_GeTe_2_ and 35 K in FePS_3_/Fe_3_GeTe_2_/FePS_3_ in comparison to the pristine Fe_3_GeTe_2_. Meanwhile, the coercive field is doubled due to the proximity coupling effect.

The quantum anomalous Hall effect (QAHE) can be made in topological insulator thin films by magnetic doping to introduce spontaneous magnetizations, such as Cr and V doped Bi_2_Te_3_ [108]. Also, spin-gapless semiconductors (SGSs) with intrinsic 2D magnetism are of great importance to the realization of topological Chern phases [3,109]. Ferromagnetic SGSs have several obvious advantages over magnetic doping in topological insulator for realizing QAHE. First, due to the experimental challenge in the uniform distribution of the magnetic impurities, the temperature at which QAHE can be realized is extremely low. Also, the ferromagnetic ground state can be sustainable against the thermal fluctuations at high temperature by a comprehensive and suitable search for SGSs. On the other hand, the Curie temperature in the diluted magnetic semiconductors can not be effectively raised without breaking the band topology. In ferromagnetic SGS the transition metal ions typically form honeycomb lattice structure including monolayers of transition-metal trihalides such as RuI_3_ [110], MnBr_3_ [111] and NiCl_3_ [112], or transition-metal oxides V_2_O_3_ [113] and Nb_2_O_3_ [114]. Kagome lattice structure is also prototypically suitable for ferromagnetic SGSs, including single layer Co_3_Sn_2_S_2_ [115], Fe_3_Sn_2_ [116], CoSn [117] and so on.

Beyond conventional spintronics, the integration of magnetic materials with topological insulators has led to the genesis of topological spintronics [118]. Hybrid structures that interface topological surface states with magnetism show promising spintronic device-related phenomena such as extremely efficient charge-to-spin conversion beyond the dissipationless charge transport from topologically protected edge states [119]. Several important observations in topological spintronics, including large spin-orbit torque, geometric Hall effect, topological antiferromagnetism and topological magnetoelectric effect, have provided great scientific opportunities toward practical goals of spintronic devices [120]. Axion insulator differs from the quantum anomalous Hall insulator in that the former is capable of the topological (near-quantized) magnetoelectric effect with broken time reversal symmetry but preserving spatial inversion symmetry [121]. To produce the axion insulating phase, an antiparallel magnetization and an opposite sign of the exchange gap should be realized on the top and bottom surfaces. Axion insulating state is prototypically characterized by a zero Hall plateau with zero Hall conductance [122]. A moderate magnetic field can drive a quantum phase transition from axion insulating phase to Chern insulator phase with zero longitudinal conductance and quantized Hall conductance [123].

### 2.4. Electric field engineering

The effective manipulation of a spin state via applying electric field plays a very important role in functionalizing 2D magnetic devices. The effective regulation of magnetism via external electric fields boosts the development of electrically controlled spintronic devices, such as voltage-controlled magnonic logic device and spin field-effect transistors with fast operation and low power consumption. However, the precise modulation of the electronic spins and the magnetism in 2D materials by external electric field has been challenging.

**Figure 6.**
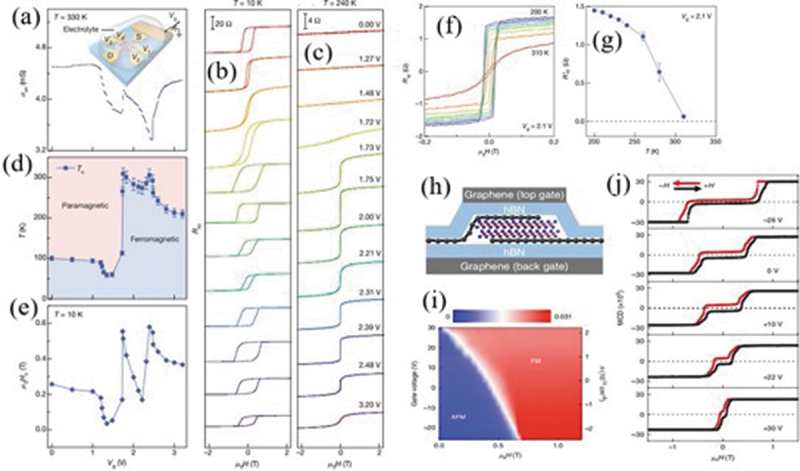
(a) Conductance as a function of gate voltage *V*_g_ measured in a trilayer FGT device. Data were obtained at *T* = 330 K. (b, c) *R_xy_* as a function of external magnetic field recorded at representative gate voltages, obtained at *T* = 10 K (b) and *T* = 240 K (c). (d) Phase diagram of the trilayer FGT sample as the gate voltage and temperature are varied. (e) Coercive field as a function of the gate voltage. (f) *R_xy_* of a four-layer FGT flake under a gate voltage of *V*_g_ = 2.1 V. (g) Remanent Hall resistance *R_xy_* as a function of temperature. Reproduced with permission from [66], Copyright 2018, Nature. (h) A schematic side view of a dual-gate bilayer CrI_3_ field-effect device. (i) Doping density–magnetic field phase diagram at 4 K. (j) MCD versus magnetic field at 4 K at representative gate voltages. Reproduced with permission from [127], Copyright 2018, Nature.

Multiple efforts have been devoted to the electric-field control of magnetism in 2D monolayers. Deng et al. [66] showed that, via ionic gating, the induced extreme charge carriers can strengthen the itinerant ferromagnetism and dramatically elevates *T*_c_ of single-layer Fe_3_GeTe_2_ above room temperature. The gate voltage can also significantly modulate the coercive field. The ferromagnetic transition temperature *T*_c_ was extracted in virtue of anomalous Hall effect measurement (Figure 6(a–g)). Wang et al. [124] showed that, via using ionic liquid and solid Si gating technology, the bipolar field effect transistor based on ferromagnetic insulating Cr_2_Ge_2_Te_6_ thin film exhibited a doping-dependent magnetism. The magneto-optical Kerr measurements demonstrated the significantly reduced saturation field and larger magnetic moment with the elevated gate voltage below the Curie temperature. Liu et al. [125] observed that the Neel-type magnetic Skyrmion spin configurations become energetic-favorable than ferromagnetic spin states as a result of applying out-of-plane electric field by breaking the inversion symmetry and inducing nontrivial Dzyaloshinskii–Moriya interaction.

Additionally, the electric-field control of interlayer magnetism in 2D layered structure have been widely reported with regard to three aspects. First, the multiple magnetic states can be induced by external electric field as a result of different patterns of layer magnetizations without breaking the magnetism of individual layer. Second, the electric field can switch the interlayer spin states and reconfigure the spin filters, indicating a great potential for magnetic logic gate device. Lastly, electric field can also induce some novel magnetic configurations, such as Skyrmion ferromagnetism in CrI_3_ [125] and spiral ferromagnetism in twisted bilayer graphene [126]. Mak et al. [127] demonstrated electrostatic doping effect on the magnetic properties of bilayer CrI_3_ by using graphene/CrI_3_ vertical heterostructure. Doping can significantly modify the interlayer exchange coupling, coercive field and *T_c_*, showing that electron/hole doping can weaken/strengthen the long-range magnetic order. The antiferromagnetic phase monotonically diminished with increasing electron doping concentration and then, totally vanished and turned into ferromagnetic phase after the elevated electron doping reaches ~2.5 × 10^13^ cm^−2^ (Figure 6(h–j)). Mak et al. [128] also demonstrated an effective regulation of magnetism via applying transverse electrical field in bilayer antiferromagnetic CrI_3_. They observed that the external electric field can induce a linear magnetoelectric effect as a result of interlayer potential difference. Xu et al. [129] used magneto-optical Kerr effect microscopy to study the electrostatic gate effect on the magnetism in CrI_3_ bilayers. They obtained electrical switching between ferromagnetic and antiferromagnetic states by applying magnetic fields near the metamagnetic transition. They also demonstrated that, due to the strong spin-layer locking, the linear gate-dependent Kerr effect signals with opposite slopes could occur with a time-reversal pair of layered antiferromagnetic states without external magnetic field. Xu et al. [130] reported the electrical switching of multiple magnetic states in four-layer CrI_3_ sandwiched between two bipolar graphene electrodes in a dual-gated field effect device. They also observed an effective gate-modulation on TMR from 17,000% to 57,000%, which is tentatively attributed to the combination of magnetic proximity effect at graphene/CrI_3_ interface and electrical field modulation on the spin-dependent tunneling as a result of Fermi level shift. San-Jose et al. [126] proposed that the relative lattice orientation between adjacent graphene layers can modulate the magnetic phases in twisted graphene bilayers. It shows a magnetic transition from lattice AFM phase to spiral FM phase for twisting graphene bilayers from 0° to a relative 120° misalignment. The vertical electric field can effectively switch the spiral FM phase and the lattice AFM phase with a relative 120° misalignment as a result of electrically tuned exchange coupling between adjacent graphene layers.

Electric field can also effectively improve the spin transport of 2D magnets via reducing spin relaxation and enhancing magnetoresistance, exhibiting a substantial potential for the practical application of spin valve device. Avsar et al. [131] fabricated a semiconducting spin valve device by encapsulating ultrathin black phosphorus (~5 nm) into hexagonal boron nitride atomic layers. They observed a long spin relaxation time of ~4 ns with spin relaxation length of ~6 µm by measuring Hanle spin precession. Liang et al. [132] showed the gate-tunable electrical spin-valve device with magnetoresistance of 1.1% in a multilayer MoS_2_ semiconducting channel on a ferromagnetic Co/MgO electrode by using a two-terminal configuration. They found that the spin relaxation is largely prevented with an enhanced spin diffusion length of 235 nm. Yang et al. [133] theoretically investigated the spin transport in spin-valve device based on Fe_3_GeTe_2_ monolayer where a high magnetoresistance of ~390% was obtained and can be significantly increased to ~510% under the electric gates.

## 3 Spintronic applications

In recent years, 2D spintronics, which utilizes the spins of polarized electrons in 2D materials for information generation, transmission and storage, has attracted a great deal of attention, and may have plenty of promising applications beyond conventional complementary metal-oxide semiconductor electronics. Especially, the manipulation of the 2D magnetism by applying external electric field and carrier doping has drawn great attention for power-saving spintronics devices. With carrier-mediated magnetism, 2D ferromagnetic/antiferromagnetic semiconductors can develop into a suit of electrically controlled spintronic devices with non-volatility and rapid-operation, such as magnetic tunnel junction, spin field-effect transistor and spin logic gate.

### 3.1. 2D magnetic tunnel junction

The 2D magnetic tunnel junction (MTJ) is composed of two separate ferromagnets as electrodes and an intermediate insulating atomic layer. Tunnel magnetoresistance is a that occurs in a MTJ via applying bias voltage. The tunneling probability depends on the electronic density of states near the Fermi energy in the ferromagnets and the thickness of the insulating barrier. When the magnetizations in two ferromagnetic electrodes are parallel, their similar distributions of the electronic density of states near the Fermi level provide more tunneling opportunities, leading to a high conductance. On the other hand, when the magnetizations of two ferromagnets are antiparallel, a mismatch of their density of states will impede the tunneling with a low conductance.

**Figure 7.**
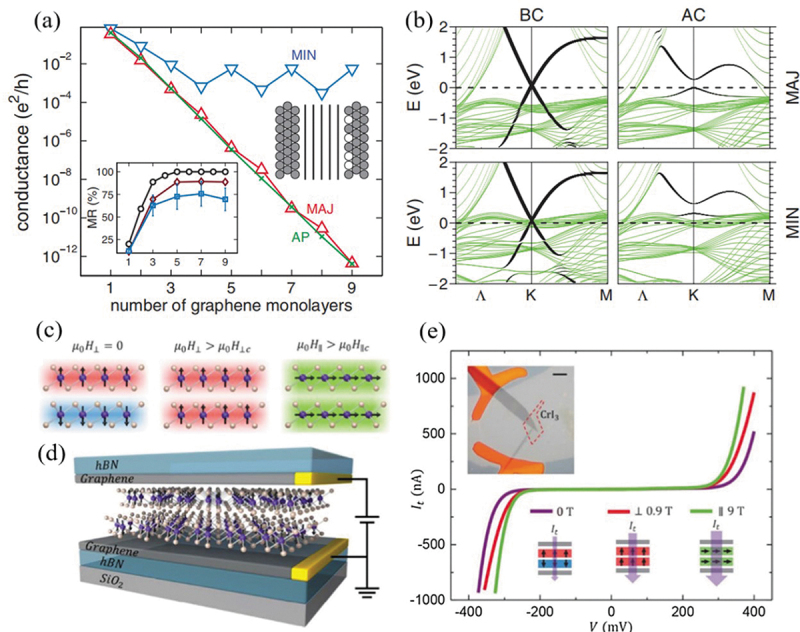
(a) Conductances $G_{P}^{min}$ (▽), $G_{P}^{maj}$ (Δ) and $G_{AP}^{\sigma }$ (*×*) of a Ni/Gr*_n_*/Ni junction as a function of the number of graphene layers *n* for ideal junctions. (b) Majority and minority spin band structures (green) of a single graphene layer absorbed upon a 13 layer (111) Ni slab for a BC configuration with *d = *3.3 Å, and an AC configuration with *d* = 2.0 Å. Reproduced with permission from [134], Copyright 2007, American Physical Society. (c) Magnetic states of bilayer CrI_3_ with different external magnetic fields. (d) Schematic illustration of a 2D spin-filter MTJ with bilayer CrI_3_ sandwiched between graphene contact. (e) Tunneling current of a bilayer CrI_3_ sf-MTJ at selected magnetic fields. Reproduced with permission from [142], Copyright 2018, Science.

Karpan et al. first reported a theoretical exploration on a MTJ based on Ni/Graphene/Co and Co/Graphene/Co by using graphene as tunneling barrier in 2007 [134]. They proposed a strong interaction between graphene and nickel slab, opening a band gap in *p_z_* band of graphene at K point in Brillouin zone for antiparallel-spin configuration. This results in a large spin polarization close to 100% and an extremely large TMR has been achieved (Figure 7(a,b)). However, the subsequently experimental observations exhibit very low TMR in 2D MTJs based on graphene layered structure, holding the highest record TMR up to 31% [118,135–138]. Scientists have devoted further effort on some other 2D materials, such as semiconducting MoS_2_ and insulating hexagonal boron nitride (h-BN), to be employed as tunnel barrier layers [139–141]. Piquemal-Banci et al. [139] fabricated Fe/h-BN/Co MTJ by using the chemical vapor deposition (CVD) method. They demonstrated that the tunneling resistance was exponentially proportional to the number of layers in h-BN. They also observed a TMR of 6% for single layer h-BN as the tunnel barrier. Zhang et al. [140] investigated Ni/MoS_2_/Ni and Co/MoS_2_/Co MTJs by their first-principles calculations, where larger TMR can be acquired by using Co as the ferromagnetic electrodes, with a maximum value of 63.86% for using five-layer MoS_2_ as the tunnel barrier and a negative value of −70.85% for single layer MoS_2_ structure.

Emerging 2D magnetic materials provide new choices and exhibit many surprising properties. Xu et al. [142] fabricated multiple spin MTJs composed of graphene/CrI_3_/graphene vdW heterostructures. They demonstrated a significantly enhanced tunneling magnetoresistance with increasing number of CrI_3_ layers. A giant TMR of 19,000% for using four-layer CrI_3_ as the tunneling barrier at low temperatures was observed (Figure 7(c–e)), which is significantly larger than other MTJs with conventional tunneling materials (e.g. MgO [143,144]). Yang et al. [145] reported the theoretical study on the spin transport properties of a MTJ based on MoSe_2_/VSe_2_/WSe_2_/VSe_2_/MoSe_2_ heterostructure by using non-equilibrium Green’s function method. Their calculations showed a large TMR of 5.6 × 10^3^%. They also observed a significantly enhanced TMR of 1.7 × 10^5^% by inserting 2 H-MoSe_2_, which strengthened the spin filtering effect at the interfaces between different atomic layers. Feng et al. [146] studied the spin transport properties of a vertical vdW MTJ based on 1 T-FeCl_2_/2 H-MoS_2_/1 T-FeCl_2_ by using metallic 1 T-MoS_2_ as electrodes. Their first-principles calculations reveal the negative differential resistance, the large spin filtering coefficient and the high TMR of 6.3 × 10^3^%. Wang et al. [147] fabricated the spin valve device based on Fe_3_GeTe_2_/h-BN/Fe_3_GeTe_2_ heterostructures. Their anomalous Hall conductivity analysis revealed that thin Fe_3_GeTe_2_ has metallic ferromagnetism with an easy-axis perpendicular to the atomic layers. The spin transport measurement showed that the spin polarization of Fe_3_GeTe_2_ is 66% at low temperature. The spin polarization evolution with increasing temperature was exactly consistent with the temperature-dependent transverse conductivity in the anomalous Hall conductivity measurement. Zhang et al. [148] also employed Fe_3_GeTe_2_ thin film as the ferromagnetic electrodes, while combining with a distinct magnetic tunnel barrier of InSe, and the TMR can reach up to ~700%. Zhou et al. [149] reported a theoretical study of the vdW MTJ based on VSe_2_/MoS_2_ heterostructure with remarkable reading and writing ability by virtue of strong spin-orbit torque effect. Their calculation results revealed a large TMR of 846% at room temperature by using non-equilibrium Green’s function. Furthermore, this team also [150] investigated the spin transport properties in 1 T-CrSe_2_/graphene/1 T-CrSe_2_ vdW MTJ by using first-principles calculations. They observed that via shifting of Fermi level by atomic substitution in the tunneling barrier with seven-layer graphene, TMR can be significantly enhanced up to about 7 × 10^3^%.

### 3.2. 2D spin field-effect transistors

Datta and Das first presented the theoretical model of 2D spin field-effect transistor (sFET) in 1990 [151]. In a 2D sFET, spin polarized current flows between ferromagnetic source and drain through a two-dimensional electron gas (2DEG) channel [151]. The vertical electric field can effectively modulate the spin polarization and magnetoresistance by tuning the spin precession arised from spin-orbit coupling in 2DEG channel. Semenov et al. [152] theoretically proposed a 2D sFET by using graphene as the channel with the ferromagnetic dielectric gate. This novel device takes advantages of long relaxation length and constant velocity of electrons in graphene in combination with the strong spin exchange in ferromagnetic gate.

Experimentally, Avsar et al. [153] reported a sFET based on dual-gated bilayer graphene with h-BN as a dielectric layer. The long spin relaxation length of ~10 μm and large carrier mobility of 2.4 × 10^4^ cm^2^ V^−1^s^− 1^ were achieved at low temperature of 2 K. The gate voltage can effectively regulate the spin relaxation time via tuning the electron doping concentration in graphene channel. They also studied the spin transport property across p–n junction via controlling the charge carriers type by the applied top/bottom gate voltage and observed a negligible spin scattering at p–n interface. Two independent groups [154,155] demonstrated that by combining the excellent spin and carrier transport properties of graphene with the strong SOC of MoS_2_, their devices composed of graphene/MoS_2_ heterostructure enable the switching of spin current between ON and OFF states by tuning the energy barrier at graphene/MoS_2_ interface with an applied gate voltage. The control of spin lifetime via electric gating was also achieved at room temperature [155]. Gong et al. [156] predicted an electric sFET based on antiferromagnetic bilayer VSe_2_ channel by first-principles calculations. The gate voltage can shift energy levels of the different layers toward opposite directions and close the band gap of singular spin-polarized states at the Fermi level, resulting in the half-metallicity with a large spin polarization. Wu et al. [157] also predicted a sFET based on bilayer VSe_2_ by using ab initio quantum transport simulations, where the spin-filter efficiency can reach up to 99%, and the conductance on-off ratio of this device is up to 10^6^.

Due to the low spin injection efficiency and fast spin relaxation in channels, it faces great difficulty to obtain a large magnetoresistance in conventional 2D sFET device. It can be enhanced significantly in the novel spin tunnel field-effect transistor (sTFET) by taking advantage of tunneling effect. Jiang et al. [158] reported a sTFET based on graphene/CrI_3_/graphene vdW heterostructure with a dual-gated configuration. By utilizing CrI_3_ atomic layers as the magnetic tunnel barrier, the vertical electric field can switch CrI_3_ layers between ferromagnetism and interlayer antiferromagnetism. Thus the devices demonstrated an ambipolar property with an arbitrary choice of high- or low-tunneling conductance. This high/low conductance ratio can be enhanced by increasing the layers number of the tunnel barrier, achieving ~400% for a four-layer MoS_2_ structure.

### 3.3. 2D spin logic gate

**Figure 8.**
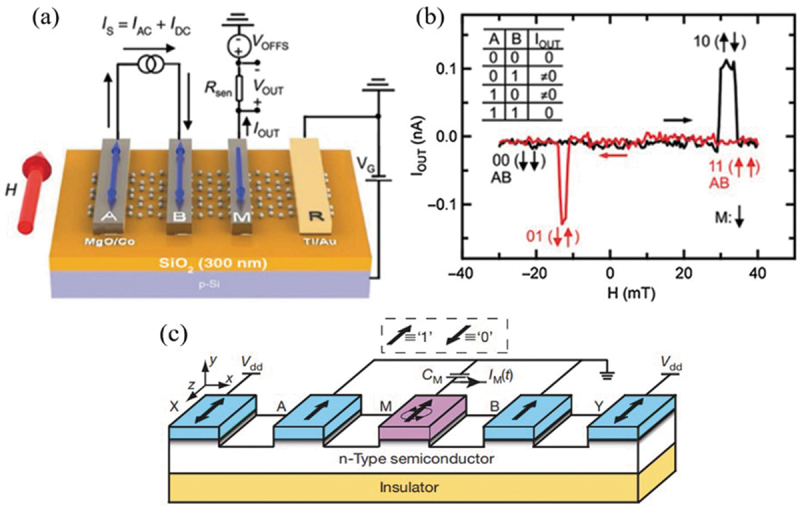
(a) Diagram of a proposed 2D XOR spin logic gate, where A, B and M are ferromagnetic electrodes on top of a spin transport channel. *I_s_* and *I_out_* denote the injection and detection currents, respectively. (b) *I_out_* measured as a function of *H*. Reproduced with permission from [160], Copyright 2016, American Physical Society. (c) Design of the reprogrammable magnetologic gate for a universal NAND operation. Reproduced with permission from [162], Copyright 2017, Nature.

Spin logic gate have been proposed as an alternative to replace giant MR effect for using as the basic logic unit for VLSI design. The spin logic structure is composed of a semiconductor channel and several ferromagnetic electrodes, in which the logic operation can be realized by injecting different spin accumulations via controlling the magnetizations in the input terminals [159–162]. As shown in Figure 8(a), the device includes the mechanically exfoliated graphene contacted by ferromagnetic cobalt (Co) electrodes A, B, and M through magnesium oxide tunnel barriers. The four different input states are realized by sweeping an external magnetic field on input electrodes A and B, resulting in a different spin accumulation in the M terminal via the spin transport through graphene channel. Therefore, the exclusive or (XOR) logic operation can be achieved, as shown in Figure 8(b) [160]. Dery et al. [162] also designed a reconfigurable magnetologic gate based on n-type semiconductor and five ferromagnetic electrodes for ‘not and’ (NAND) operation, which can have fast and effective logic operations in a noisy and room-temperature environment (Figure 8(c)). Subsequently, the studies have been extended from the theoretical design to the experimental explorations of 2D spin logic gates [163–165]. Kim et al. [163] fabricated both nonvolatile logic gate meta-devices with two input terminals composed of graphene, several ferroelectric polymer layers and meta-atoms. Various logic operations (e.g. XOR, AND and OR) can be achieved in their devices at room temperature. Two-bit digital-to-analogue conversion was also demonstrated by four levels of optical analogue states determined by two digital input states.

### 3.4. 2D spin diode

**Figure 9.**
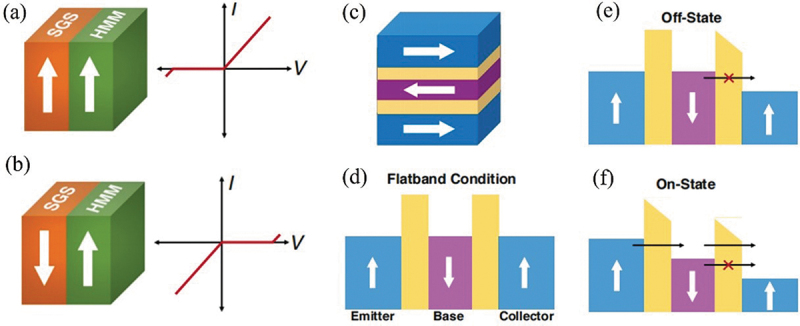
(a) A schematic representation of the HMM-SGS junction for parallel orientation of the magnetization directions of the electrodes and the corresponding current–voltage (*I*–*V*) curves. (b) The same as (a) for the antiparallel orientation of the magnetization directions of the electrodes. Reproduced with permission from [168], Copyright 2020, American Physical Society. (c) Schematic representation of the magnetic tunnel transistor. (d) Band diagram of the MTT under flatband condition, (e) the OFF-state, and (f) the ON-state. Reproduced with permission from [169], Copyright 2019, American Chemical Society.

Among 2D magnets, spin-gapless semiconductors (SGSs) [166,167] are promising materials for the realization of ultra-fast and ultra-low-power spintronic devices due to their high mobility and tunable electrical properties intertwined with low energy spin-polarized Dirac dispersion. In additional to magnetic tunnel junctions, spin field-effect transistors and spin logic gate, 2D Spin diode is another useful spintronic application by assembling SGSs and half metal magnets (HMMs) with intrinsic magnetism. Sasioglu et al. theoretically proposed a novel spin diode based on a two terminal junction with SGSs and HMMs in absence of the tunnel barrier [168]. The finite forward bias can induce Ohmic contact at SCSs-HMMs interface while the reverse current is forbidden, demonstrating a linear current-voltage characteristics, as shown in Figure 9(a,b). Sasioglu et al. also designed the magnetic tunneling transistor with the three-terminal structure of HMMs–SGSs–HMMs (emitter-base-collector) [169]. As shown in Figure 9(c–f), the on and off states can be effectively switched by modulating the voltage of the gate terminal, demonstrating a current–voltage characteristic in both directions similar to the conventional bipolar junction transistors [169]. Also, Eisenstein et al. [170] theoretically demonstrated that, by applying a strong out-of-plane magnetic field, quantum hall spin diode can be achieved in double layer two-dimensional electron systems with antiparallel interlayer spin configurations at low temperature. The nearly ideal spin-diodelike behavior is attributed to the essentially complete spin polarization of the valence Landau level in each atomic layer.

## 4 Challenges and opportunities

Firstly, it is always urgently needed and promising for discovering new members of 2D magnetic materials with high-transition temperatures, high coercive field and good environmental stability. Most currently available 2D magnets possess low phase transition temperature far below room temperature [7]. Meanwhile, the stability is another great challenge that most 2D materials with atomic thickness are susceptible to temperature effect, oxygenation, moisture and other chemical corrosion [20]. Also, the exploration of low-dimensional magnetic topological insulators with topological spin textures and intrinsic chiral helimagnetism is crucial for developing their practical applications in dissipationless topological electronics and topological quantum computation.

Secondly, it is imperative to develop some effective and controllable synthesis methods of 2D magnetic materials fitting to different practical purposes. At present, the most common and conventional fabrication method is mechanical exfoliation from their pristine crystals [7]. Although this technique possesses the virtues of simplicity with low cost, solution processability, high quality, ultrathin structure and easy hybridization for versatile functionalities, which are favorable for fundamental scientific studies; however, we cannot ignore the negatives, such as low yield, random distribution, uncontrollable thickness, and tiny size, which could impede its practicality of device production and engineering [171]. By inspiration from the abundant experimental experiences on conventional low-dimensional non-magnetic materials, we can take advantages of multiple synthesis methods, including chemical vapor deposition (CVD), molecular beam epitaxy (MBE), physical vapor deposition (PVD), etc., on the novel 2D magnetic materials.

Lastly, there have been made great breakthroughs in 2D spintronic devices, for example, a long spin diffusion length of 30 mm with long spin lifetimes over 12 ns at room temperature in spin valve devices based on graphene/hBN heterostructure [87] and high TMR up to 1.9 × 10^4^% in MTJs composed of graphene/CrI_3_/graphene vdW heterostructures [93]. However, it is noteworthy that the TMR is still too low to be applied for the basic logic unit matched with commercialized complementary metal-oxide semiconductor electronics with typical on/off ratio above 10^5^ in VLSI design. In other words, the development of practical 2D spintronic devices are still in its early states of theoretical- and experimental-explorations those are very far away from the mature industrial magnetics and electronics. Recently, it has been shown that electric-field induced Rashba effect is a key phenomenon for overcoming Boltzmann tyranny via topological quantum field effect and thus leading to energy-efficient switching devices by reducing subthreshold swing [172]. Since the intertwining between ferromagnetic exchange interaction, spin polarization, and the Rashba effect plays a central role in 2D magnetic materials, it can also be highlighted that such a topological quantum field effect in 2D magnetic materials may prove a promising ingredient for low-voltage spintronic devices.

## 5 Conclusion

A comprehensive review has been focused on the recent scientific advances on the manipulation strategies, such as strain-, doping-, structural- and electric field-engineering, on a wide variety of emerging 2D magnetic materials. Also, great breakthroughs have been made toward practical 2D spintronic applications, including spin tunneling junctions, spin field-effect transistors, and spin logic gate, etc. However, practical application of 2D spintronic devices still necessitate overcoming great obstacles, including discovery of new 2D magnetic materials with high Curie temperature, development of more effective and controllable synthesis methods and enhancement of TMR for spin MTJs applied for VLSI design. The exploration of novel 2D magnetic materials, the improvement of theoretical models, and the development of experimental technologies all offer broaden opportunities for overcoming the current challenges in their practical application, accelerated industrialization and promising commercialization in information technologies.
